# Clustering Coefficients for Correlation Networks

**DOI:** 10.3389/fninf.2018.00007

**Published:** 2018-03-15

**Authors:** Naoki Masuda, Michiko Sakaki, Takahiro Ezaki, Takamitsu Watanabe

**Affiliations:** ^1^Department of Engineering Mathematics, University of Bristol, Bristol, United Kingdom; ^2^School of Psychology and Clinical Language Sciences, University of Reading, Reading, United Kingdom; ^3^Research Institute, Kochi University of Technology, Kochi, Japan; ^4^PRESTO, Japan Science and Technology Agency, Kawaguchi, Japan; ^5^Institute of Cognitive Neuroscience, University College London, London, United Kingdom

**Keywords:** network neuroscience, clustering coefficient, functional connectivity, partial correlation, partial mutual information, aging

## Abstract

Graph theory is a useful tool for deciphering structural and functional networks of the brain on various spatial and temporal scales. The clustering coefficient quantifies the abundance of connected triangles in a network and is a major descriptive statistics of networks. For example, it finds an application in the assessment of small-worldness of brain networks, which is affected by attentional and cognitive conditions, age, psychiatric disorders and so forth. However, it remains unclear how the clustering coefficient should be measured in a correlation-based network, which is among major representations of brain networks. In the present article, we propose clustering coefficients tailored to correlation matrices. The key idea is to use three-way partial correlation or partial mutual information to measure the strength of the association between the two neighboring nodes of a focal node relative to the amount of pseudo-correlation expected from indirect paths between the nodes. Our method avoids the difficulties of previous applications of clustering coefficient (and other) measures in defining correlational networks, i.e., thresholding on the correlation value, discarding of negative correlation values, the pseudo-correlation problem and full partial correlation matrices whose estimation is computationally difficult. For proof of concept, we apply the proposed clustering coefficient measures to functional magnetic resonance imaging data obtained from healthy participants of various ages and compare them with conventional clustering coefficients. We show that the clustering coefficients decline with the age. The proposed clustering coefficients are more strongly correlated with age than the conventional ones are. We also show that the local variants of the proposed clustering coefficients (i.e., abundance of triangles around a focal node) are useful in characterizing individual nodes. In contrast, the conventional local clustering coefficients were strongly correlated with and therefore may be confounded by the node's connectivity. The proposed methods are expected to help us to understand clustering and lack thereof in correlational brain networks, such as those derived from functional time series and across-participant correlation in neuroanatomical properties.

## 1. Introduction

Networks have been proven to be a useful language to understand structural and functional properties of the brain. The research field is collectively called network neuroscience (Bassett and Sporns, 2017). Initial studies in network neuroscience revealed that brain networks on various spatial scales have properties common to other biological and non-biological networks, such as the small-world property and community structure. More recent studies tend to depend on the availability of new tools to record data with, look at other properties of brain networks such as network hubs, rich clubs and economic efficiency, and endeavor into the analysis of impaired brains (Bullmore and Sporns, 2009; Sporns, 2011; Fornito et al., 2013; Stam, 2014; Bassett and Sporns, 2017).

In this article, we focus on a measure which has often been applied to brain (and other) networks: clustering coefficient (Watts and Strogatz, 1998). The clustering coefficient quantifies the abundance of connected triangles in a network. In network neuroscience, the clustering coefficient has been shown to be a useful quantity for understanding function-structure associations in the brain for at least the following two reasons. First, it is one of the two building blocks with which to measure the small-worldness of a network; small-world networks are those having a large clustering coefficient and a small shortest path length between two nodes (such as regions of interest; ROIs) on average (Watts and Strogatz, 1998; Bullmore and Sporns, 2009). Brain networks are usually small-world networks in this sense (Achard et al., 2006; Bassett and Bullmore, 2006). Loss of small-worldness is a signature of, for example, Alzheimer disease (Supekar et al., 2008; Brier et al., 2014) and schizophrenia (Liu et al., 2008). Second, the abundance of connected triangles around a given node, which is measured by local variants of the clustering coefficient, informs us of other structure and functions of networks, namely, community structure (Radicchi et al., 2004; Palla et al., 2005) and local efficiency (Latora and Marchiori, 2001). Both community structure and local efficiency are often measured for brain networks (Achard and Bullmore, 2007; Bullmore and Sporns, 2009; Rubinov and Sporns, 2010, 2011); for example, community structure of functional brain networks is less pronounced in childhood-onset schizophrenia than controls (Alexander-Bloch et al., 2010).

However, the current measurement of the clustering coefficient can be easily fooled when it is applied to correlational brain/neuronal networks, where the connectivity between two nodes is defined by Pearson correlation and potentially some other correlation indices. Such correlational brain networks are often built on the basis of a correlation measure between two ROIs such as the pairwise correlation between time-dependent blood oxygen level-dependent (BOLD) signals obtained from functional magnetic resonance imaging (fMRI) or neural signals obtained from electroencephalogram (EEG) and magnetoencephalogram (MEG) (Bullmore and Sporns, 2009; Bassett and Sporns, 2017). Correlational networks are also employed to construct structural networks of the brain, where an edge between two ROIs is calculated as the across-participant correlation in the cortical thickness (Alexander-Bloch et al., 2013; Evans, 2013). A naive application of network analysis tools, including the clustering coefficient, to such correlation networks can go awry due to the following reasons.

First, a network derived from a correlation matrix tends to have many triangles owing to the so-called indirect paths, i.e., a correlation between nodes *i* and *j* and one between *i* and ℓ result in a correlation between *j* and ℓ even when there is no direct relationship between *j* and ℓ (Adachi et al., 2012; Zalesky et al., 2012). This mathematical property raises the clustering coefficient values. The same pseudo-correlation effect also automatically produces an inflated correlation between the connectivity of node *i* and the local clustering coefficient (i.e., which refers to the abundance of triangles around a particular node *i* and has been used for characterizing individual ROIs Sporns and Zwi, 2004; Achard et al., 2006; He et al., 2007; Alexander-Bloch et al., 2010; Lynall et al., 2010; Power et al., 2010; van den Heuvel et al., 2010; van den Heuvel and Sporns, 2011; Wee et al., 2011; Fornito et al., 2012; Tijms et al., 2013; Sala-Llonch et al., 2014) as we will show (section 3.5). One remedy is to use appropriate null models (Zalesky et al., 2012), which respect the natural constraints imposed on correlation matrices including a large clustering coefficient value even in the case of networks generated at random. Nevertheless, this solution does not address the issue of the threshold value, which we will discuss below. The partial correlation matrix is a method of choice for removing pseudo-correlation between ROIs that is present in networks based on the Pearson correlation matrix. However, estimation of the partial correlation matrix is difficult, particularly when the number of image volumes is relatively small as compared to the number of ROIs, which is typical of fMRI experiments (Schäfer and Strimmer, 2005; Ryali et al., 2012; Brier et al., 2015).

Second, to create a network, we conventionally threshold on the correlation value to dichotomize the presence or absence of an edge between each pair of ROIs. However, the choice of the threshold is arbitrary (Rubinov and Sporns, 2010, 2011; De Vico Fallani et al., 2014; Garrison et al., 2015) and results of graph-theoretical analyses often depend on the choice of the threshold (Zalesky et al., 2012; Garrison et al., 2015; Jalili, 2016). Specifically, clustering coefficient values considerably depend on the threshold value (Zalesky et al., 2012; Garrison et al., 2015). One can avoid thresholding by using weighted networks, i.e., networks with weighted edges (Rubinov and Sporns, 2010, 2011). There are several definitions of clustering coefficient for weighted networks (Barrat et al., 2004; Onnela et al., 2005; Zhang and Horvath, 2005; Saramäki et al., 2007; Rubinov and Sporns, 2010, 2011; Costantini and Perugini, 2014; Wang et al., 2017). However, it is unclear how the weighted network approach should deal with negatively weighted edges; most network analysis tools including the clustering coefficient assume non-negative edges (Newman, 2010). An interesting possibility is to separately analyse networks composed of positive edges and those composed of negative edges, and then to combine the measurements obtained from the two types of networks (Rubinov and Sporns, 2011). However, there seems to be no consensus regarding the treatment of negatively signed edges (Sporns and Betzel, 2016).

In the present study, we develop two clustering coefficients tailored to correlation matrices. The first type of clustering coefficient is based on three-way partial correlation coefficient. The second type is based on partial mutual information. Partial mutual information is a nonlinear correlation measure, which is defined as the conventional mutual information between two random variables but conditioned on other variables (Frenzel and Pompe, 2007). These clustering coefficients are expected to overcome some of the aforementioned difficulties. First, they discount the effect of indirect paths to quantify association between two neighbors of a node given the activity of the focal node. In this manner, we avoid both the problem of pseudo-correlation in ordinary correlation matrices and computational issues in the calculation of partial correlation matrices. Second, as in the case of the clustering coefficients for weighted networks, our clustering coefficients do not use thresholding on the correlation value. Third, we measure how far the realized pairwise correlation value is (no matter positive or negative) from the correlation anticipated by the presence of indirect paths. Although this treatment does not solve the problem of negative edges, we intend to use the information contained in negative as well as positive edges in this manner. For a proof of concept, we apply the proposed clustering coefficient indices to fMRI data obtained from healthy subjects with a wide range of age. We show that the clustering coefficients are negatively correlated with the age. This observation is in general less pronounced with the conventional clustering coefficient measures, although decline in the clustering coefficient with ageing should not be regarded as a ground truth in light of the literature (Wang et al., 2010; Matthäus et al., 2012; Zhu et al., 2012; Brier et al., 2014; Liu et al., 2014; Sala-Llonch et al., 2014; Knyazev et al., 2015; Grady et al., 2016). We also show that the local clustering coefficients at specific ROIs provide information orthogonal to the mere strength of connectivity and that their association with the participant's age is independent of brain systems.

## 2. Methods

### 2.1. Functional connectivity

We used *N*_ROI_ = 30 regions of interest (ROIs) whose coordinates were determined in a previous study (Fair et al., 2009). Note that we excluded the four cerebellar ROIs out of the 34 ROIs. The system of the 30 ROIs contained the default mode network (DMN; 12 ROIs), cingulo-opercular network (CON; 7 ROIs) and fronto-parietal network (FPN; 11 ROIs).

Denote by ρ(*i, j*) the Pearson correlation coefficient between the BOLD signals at two ROIs *i* and *j* (1 ≤ *i, j* ≤ *N*_ROI_). We primarily use ρ(*i, j*) as a measure of functional connectivity between ROIs. However, we will discount the effect of indirect paths, which is present when the edge between ROIs *i* and *j* is solely determined by ρ(*i, j*), by defining new clustering coefficients (section 2.5).

For comparison purposes, we will also examine conventional clustering coefficients for networks (sections 2.3, 2.4), which are applied to the Pearson correlation matrix and the partial correlation matrix. The partial correlation matrix, which we use as a benchmark, is an alternative measure of functional connectivity (Salvador et al., 2005; Marrelec et al., 2006), and its (*i, j*) element is estimated by ${\bar{\rho }}^{\text{partial}}\left(i,j\right)=-{\text{cov}}^{-1}\left(i,j\right)/\sqrt{{\text{cov}}^{-1}\left(i,i\right){\text{cov}}^{-1}\left(j,j\right)}$, where cov denotes the covariance matrix (Whittaker, 1990). It should be noted that ρ(*i, j*) = ρ(*j, i*) and ${\bar{\rho }}^{\text{partial}}\left(i,j\right)\;=\;{\bar{\rho }}^{\text{partial}}\left(j,i\right)$. We interchangeably use node and ROI in the following.

### 2.2. Average functional connectivity

We used the following two indices of average functional connectivity: the pairwise Pearson correlation coefficient averaged over all pairs of ROIs, denoted by *s*, and the same average but only over the ROI pairs having the non-negative ρ(*i, j*) values, denoted by *s*^+^. The introduction of *s*^+^ is motivated by the observation that the interpretation of negative correlation coefficients remains difficult (Fox et al., 2009; Murphy et al., 2009; Rubinov and Sporns, 2011; Fornito et al., 2013).

### 2.3. Clustering coefficients for unweighted networks

In this section and the next, we explain the previously proposed clustering coefficients for unweighted and weighted networks based on the Pearson correlation coefficient, ρ(*i, j*). Those based on the partial correlation coefficient, ${\bar{\rho }}^{\text{partial}}\left(i,j\right)$, are analogously calculated.

To construct an unweighted functional network, we lay an edge between nodes *i* and *j* (1 ≤ *i* ≠ *j* ≤ *N*) if and only if ρ(*i, j*) ≥ θ, where θ is a pre-determined threshold. The generated network is undirected. We denote the adjacency matrix of the network by *A* = (*a*_*ij*_), where 1 ≤ *i, j* ≤ *N*_ROI_. In other words, *a*_*ij*_ = 1 if (*i, j*) is an edge and *a*_*ij*_ = 0 otherwise. The clustering coefficient represents the abundance of connected triangles in a network (Watts and Strogatz, 1998). The local clustering coefficient of node *i* is defined by

(1) $$\begin{array}{l} C_{i}^{\text{unw}}=\frac{\left(\text{Number of connected triangles including node }i\right)}{k_{i}\left(k_{i}-1\right)/2}\\ \;=\frac{\sum_{\begin{array}{c} 1\leq j<\ell \leq N_{\text{ROI}}\\ j,\ell \neq i \end{array}}a_{ij}a_{i\ell }a_{j\ell }}{k_{i}\left(k_{i}-1\right)/2}, \end{array}$$

where $k_{i}\;=\;\overset{N_{\text{ROI}}}{\underset{j=1}{\sum }}\;a_{ij}\;=\;\overset{N_{\text{ROI}}}{\underset{j=1}{\sum }}\;a_{ji}$ is the degree of node *i*, i.e., the number of edges to which node *i* is adjacent. The denominator on the right-hand side of Equation (1) represents the largest possible number of triangles to which node *i* belongs. Note that $0\leq C_{i}^{\text{unw}}\leq 1$ (1 ≤ *i* ≤ *N*_ROI_) and that $C_{i}^{\text{unw}}$ is undefined if *k*_*i*_ = 0 or 1. The global clustering coefficient for the entire network, denoted by *C*^unw^, is given by the average of $C_{i}^{\text{unw}}$ over all nodes. We exclude the nodes with *k*_*i*_ ≤ 1 from the calculation of *C*^unw^. Note that 0 ≤ *C*^unw^ ≤ 1. Similar to other types of networks, most brain networks, anatomical or functional, have large values of *C*^unw^ as compared to randomized networks (Bullmore and Sporns, 2009; Bassett and Sporns, 2017).

### 2.4. Clustering coefficients for weighted networks

One can define a weighted functional network by regarding ρ(*i, j*) as the weight of edge (*i, j*). Because we do not have established methods to deal with negatively weighted edges (but see Rubinov and Sporns, 2011) and it is common to discard edges with a negative ρ(*i, j*) value (Rubinov and Sporns, 2010; Kaiser, 2011), the weighted adjacency matrix is given by *w*_*ij*_ = ρ(*i, j*) if ρ(*i, j*) > 0 and *w*_*ij*_ = 0 otherwise. As benchmarks, we consider three variants of weighted clustering coefficient commonly used in the literature (Saramäki et al., 2007; Rubinov and Sporns, 2010, 2011; Wang et al., 2017). We denote by (*a*_*ij*_) the adjacency matrix of the unweighted network obtained by ignoring the edge weight in the weighted network. In other words, we set *a*_*ij*_ = 1 if *w*_*ij*_ > 0 (equivalently, ρ(*i, j*) > 0) and *a*_*ij*_ = 0 otherwise.

The local clustering coefficient of node *i* proposed by Barrat et al. (2004) is given by

(2) $$\begin{array}{l} C_{i}^{\text{wei},\text{B}}=\frac{1}{s_{i}\left(k_{i}-1\right)}\underset{\begin{array}{c} 1\leq j,\ell \leq N_{\text{ROI}}\\ j,\ell \neq i \end{array}}{\sum }\frac{w_{ij}+w_{i\ell }}{2}a_{ij}a_{i\ell }a_{j\ell }, \end{array}$$

where $s_{i}\;=\;\overset{N_{\text{ROI}}}{\underset{j=1}{\sum }}\;w_{ij}$ is the node strength (i.e., weighted degree). It should be noted that *a*_*ij*_*a*_*iℓ*_*a*_*jℓ*_ = 1 if and only if nodes *i*, *j* and ℓ form a triangle in the unweighted network; *a*_*ij*_*a*_*iℓ*_*a*_*jℓ*_ = 0 otherwise. The average of $C_{i}^{\text{wei},\text{B}}$ over all nodes defines the global weighted clustering coefficient denoted by *C*^wei,B^.

The local clustering coefficient proposed by Onnela et al. (2005), which is implemented in the Brain Connectivity Toolbox (Rubinov and Sporns, 2010), is given by

(3) $$\begin{array}{l} C_{i}^{\text{wei},\text{O}}=\frac{1}{k_{i}\left(k_{i}-1\right)}\underset{\begin{array}{c} 1\leq j,\ell \leq N_{\text{ROI}}\\ j,\ell \neq i \end{array}}{\sum }\frac{{\left(w_{ij}w_{i\ell }w_{j\ell }\right)}^{1/3}}{\max_{i\prime j^{\prime }}w_{i\prime j^{\prime }}}. \end{array}$$

Factor $\underset{i\prime j^{\prime }}{\max}w_{i\prime j^{\prime }}$ normalizes $C_{i}^{\text{wei},\text{O}}$ between 0 and 1 and prevents it from scaling when the scale of *w*_*ij*_ is changed (i.e., when *w*_*ij*_ for all 1 ≤ *i, j* ≤ *N*_ROI_ is multiplied by the same constant). The corresponding global clustering coefficient, denoted by *C*^wei,O^, is given by the average of $C_{i}^{\text{wei},\text{O}}$ over all nodes.

The local clustering coefficient proposed by Zhang and Horvath (2005) is written as (Saramäki et al., 2007)

(4) $$\begin{array}{l} C_{i}^{\text{wei},\text{Z}}=\frac{1}{\max_{i\prime j^{\prime }}w_{i\prime j^{\prime }}}\frac{\sum_{\begin{array}{c} 1\leq j,\ell \leq N_{\text{ROI}}\\ j,\ell \neq i \end{array}}w_{ij}w_{i\ell }w_{j\ell }}{\sum_{\begin{array}{c} 1\leq j,\ell \leq N_{\text{ROI}}\\ j,\ell \neq i;j\neq \ell \end{array}}w_{ij}w_{i\ell }}. \end{array}$$

The corresponding global clustering coefficient, denoted by *C*^wei,Z^, is given by the average of $C_{i}^{\text{wei},\text{Z}}$ over all nodes.

### 2.5. Our proposal: clustering coefficients tailored to correlation matrices

**Figure 1:**
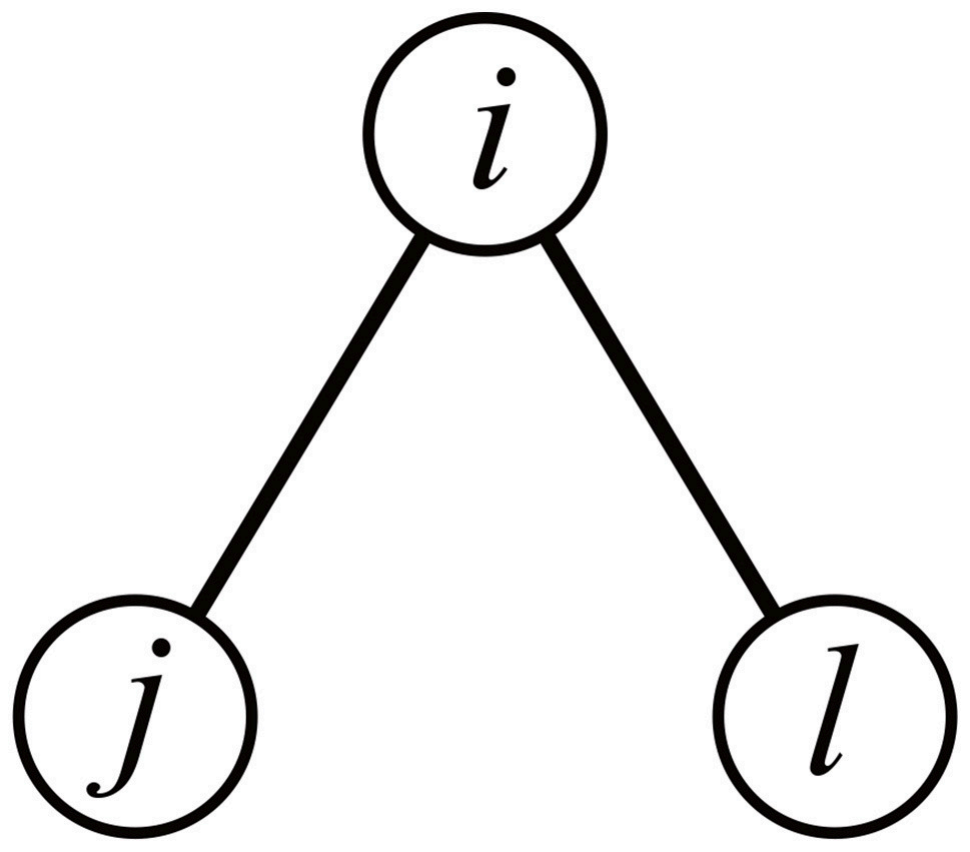
Schematic of the indirect path between nodes *j* and ℓ through node *i*.

We propose two clustering coefficient measures for correlation matrices (*C*^cor,A^ and *C*^cor,M^). Both of them discount correlation between ROIs *j* and ℓ that is expected from the correlation between ROIs *i* and *j* and that between *i* and ℓ, i.e., indirect path between *j* and ℓ through *i* (Figure 1) (Zalesky et al., 2012). One measure uses the three-way partial correlation coefficient and the other measure uses the partial mutual information.

The three-way partial correlation coefficient between ROIs *j* and ℓ controlling for the influence of ROI *i*, denoted by ρ^partial^(*j*, ℓ|*i*), is defined by Whittaker (1990)

(5) $$\begin{array}{l} \rho^{\text{partial}}\left(j,\ell |i\right)=\frac{\rho \left(j,\ell \right)-\rho \left(i,j\right)\rho \left(i,\ell \right)}{\sqrt{1-\rho^{2}\left(i,j\right)}\sqrt{1-\rho^{2}\left(i,\ell \right)}}. \end{array}$$

Equation (5) indicates that ROIs *i* and *j* would be correlated with an amount ρ(*i, j*)ρ(*i*, ℓ) by default owing to the indirect path between *j* and ℓ through *i* (e.g., Zalesky et al., 2012). Deviations of ρ(*j*, ℓ) from ρ(*i, j*)ρ(*i*, ℓ) quantify the tendency that *j* and ℓ are more strongly or weakly connected than is expected from the presence of an indirect path between *j* and ℓ through *i*. Based on this observation, we define a first variant of the clustering coefficient as follows.

It is difficult to interpret negative correlation values in functional connectivity data (Fox et al., 2009; Murphy et al., 2009; Rubinov and Sporns, 2011; Smith et al., 2011; Sporns, 2011; Fornito et al., 2013). Therefore, we assume that any deviation of ρ(*j*, ℓ) from ρ(*i, j*)ρ(*i*, ℓ) caused by the effect of *i*, irrespective of whether it is positive or negative, contributes to the local clustering coefficient at *i*. We define the local clustering coefficient for ROI *i*, denoted by $C_{i}^{\text{cor},\text{A}}$ (superscript A standing for the absolute value), as

(6) $$\begin{array}{l} C_{i}^{\text{cor},\text{A}}=\frac{\sum_{\begin{array}{c} 1\leq j<\ell \leq N_{\text{ROI}}\\ j,\ell \neq i \end{array}}|\rho \left(i,j\right)\rho \left(i,\ell \right)\rho^{\text{partial}}\left(j,\ell |i\right)|}{\sum_{\begin{array}{c} 1\leq j<\ell \leq N_{\text{ROI}}\\ j,\ell \neq i \end{array}}|\rho \left(i,j\right)\rho \left(i,\ell \right)|}. \end{array}$$

In other words, $C_{i}^{\text{cor},\text{A}}$ is a weighted average of the absolute value of the partial correlation over pairs of *j* and ℓ. We have employed the weight |ρ(*i, j*)ρ(*i*, ℓ)| for averaging because a high clustering around ROI *i* should imply strong association between ROIs *j* and ℓ (in the sense of partial correlation) when *i* and *j* are strongly connected and *i* and ℓ are. We have used ρ^partial^(*j*, ℓ|*i*) instead of ${\bar{\rho }}^{\text{partial}}\left(j,\ell \right)$, i.e., the partial correlation between *j* and ℓ controlling for the effect of the other *N*_ROI_−2 ROIs, to make $C_{i}^{\text{cor},\text{A}}$ a locally calculated quantity as is the case for the clustering coefficients for networks (e.g., $C_{i}^{\text{und}}$, $C_{i}^{\text{wei},\text{B}}$, $C_{i}^{\text{wei},\text{O}}$ and $C_{i}^{\text{wei},\text{Z}}$). The corresponding global clustering coefficient, denoted by *C*^cor,A^, is given by the average of $C_{i}^{\text{cor},\text{A}}$ over all nodes. Note that $0\leq C_{i}^{\text{cor},\text{A}}\leq 1\;\left(1\leq i\leq N_{\text{ROI}}\right)$ and 0 ≤ *C*^cor,A^ ≤ 1.

We also use another definition of the clustering coefficient based on the partial mutual information, which is a nonlinear correlation measure (Frenzel and Pompe, 2007). By definition, the mutual information is nonnegative and invariant under flipping of the sign of the random variable. We use the partial mutual information between ROIs *j* and ℓ conditioned on ROI *i* in place of ρ^partial^(*j*, ℓ|*i*) to define the second variant of the local clustering coefficient for correlation matrices, denoted by $C_{i}^{\text{cor},\text{M}}$ (superscript M standing for the mutual information).

The partial mutual information is defined as

(7) $$\begin{array}{l} I\left(X_{j},X_{\ell }|X_{i}\right)=h\left(X_{j},X_{i}\right)+h\left(X_{\ell },X_{i}\right)-h\left(X_{i}\right)-h\left(X_{j},X_{\ell },X_{i}\right), \end{array}$$

where *X*_*i*_, *X*_*j*_ and *X*_ℓ_ are the random variables on ROIs *i*, *j* and ℓ, respectively, and *h* is the (joint) entropy. For example, $h\left(X_{i}\right)=-\underset{x}{\sum }p\left(x\right)\log_{2}p\left(x\right)$, where *p*(*x*) is the probability that *X*_*i*_ = *x*, and $h\left(X_{j},X_{i}\right)=-\underset{x,x^{\prime }}{\sum }p\left(x,x^{\prime }\right)\log_{2}p\left(x,x^{\prime }\right)$, where *p*(*x, x*′) is the probability that $\left(X_{j},X_{i}\right)=\left(x,x^{\prime }\right)$. By assuming that the BOLD signals at ROIs *i*, *j* and ℓ obey a multivariate Gaussian distribution, one obtains the entropy values in Equation (7) as follows (Rieke et al., 1999; Cover and Thomas, 2006; Frenzel and Pompe, 2007):

(8) $$\begin{array}{l} h\left(X_{\alpha_{1}},\ldots ,X_{\alpha_{d}}\right)=\frac{d}{2}\left(1+\ln\;2\pi \right)+\frac{1}{2}\ln\;\det{\text{cov}}^{\prime }, \end{array}$$

where *d* is the number of random variables and ${\text{cov}}^{\prime }=\left({\text{cov}}_{ij}^{\prime }\right)$ is the *d* × *d* covariance matrix constructed by *X*_α_1__, …, *X*_α_*d*__, i.e., ${\text{cov}}_{ij}^{\prime }=\text{E}\left[X_{\alpha_{i}}X_{\alpha_{j}}\right]$, where E[·] represents the expectation. By substituting Equation (8) in Equation (7) and setting ${\text{cov}}_{ij}^{\prime }=\rho \left(i,j\right)$, we obtain

(9) $$\begin{array}{l} I(X_{j},X_{\ell }|X_{i})=\frac{1}{2}[\ln\left(1-\rho^{2}(i,j)\right)+\ln\left(1-\rho^{2}(i,\ell )\right)\\ \;-\ln(1-\rho^{2}(i,j)-\rho^{2}(i,\ell )-\rho^{2}(j,\ell )\\ \;+\;2\rho (i,j)\rho (i,\ell )\rho (j,\ell ))]. \end{array}$$

Using the partial mutual information, we define

(10) $$\begin{array}{l} C_{i}^{\text{cor},\text{M}}=\frac{\sum_{\begin{array}{c} 1\leq j<\ell \leq N_{\text{ROI}}\\ j,\ell \neq i \end{array}}|\rho \left(i,j\right)\rho \left(i,\ell \right)|I\left(X_{j},X_{\ell }|X_{i}\right)}{\frac{1+\ln\;2\pi }{2}\sum_{\begin{array}{c} 1\leq j<\ell \leq N_{\text{ROI}}\\ j,\ell \neq i \end{array}}|\rho \left(i,j\right)\rho \left(i,\ell \right)|}. \end{array}$$

The denominator normalizes the $C_{i}^{\text{cor},\text{M}}$ value to range between 0 and 1. The corresponding global clustering coefficient, denoted by *C*^cor,M^, is given by the average of $C_{i}^{\text{cor},\text{M}}$ over all nodes.

As a robustness test, we also examined variants of these clustering coefficients constrained to only positive triangles or negative triangles. We define *C*^cor,A,+^ by restricting the enumeration of triangles in the calculation of *C*^cor,A^ to the positive triangles. In other words, we restrict the summation on the numerator and denominator of Equation (6) to *j* and ℓ satisfying ρ(*i, j*), ρ(*i*, ℓ), ρ(*j*, ℓ) > 0. We similarly define *C*^cor,A,−^, *C*^cor,M,+^ and *C*^cor,M,−^. We removed six participants from the calculation of *C*^cor,A,−^ and *C*^cor,M,−^. This is because, for these participants, there was at least one ROI *i* at which there was no triangle with ρ(*i, j*), ρ(*i*, ℓ), ρ(*j*, ℓ) < 0, rendering *C*^cor,A,−^ and *C*^cor,M,−^ undefined.

We provided C++ code for calculating the proposed clustering coefficients on Github (https://github.com/naokimas/clustering-corr-mat).

### 2.6. H-Q-S algorithm

As a null model of the covariance matrix, we employed the Hirschberger-Qu-Steuer (H-Q-S) algorithm (Hirschberger et al., 2007). As recent fMRI data analysis has demonstrated, the H-Q-S algorithm is a more suitable null model than conventional null models in which the topology is randomized (Zalesky et al., 2012; Hosseini and Kesler, 2013). The H-Q-S algorithm preserves the mean of the diagonal elements, the mean of the off-diagonal elements and the variance of the off-diagonal elements of the given covariance matrix. From the fMRI data of each participant, we obtained the covariance matrix in the course of calculating the functional connectivity, which is the correlation matrix. Based on this covariance matrix, we generated random covariance matrices using H-Q-S algorithm. We then converted the generated random covariance matrices into correlation matrices, which were used as randomized functional connectivity matrices. We did not implement a fine-tuned heuristic variant proposed in Zalesky et al. (2012).

Denote by μ_on_ the average of the diagonal elements of the covariance matrix over the *N*_ROI_ diagonal elements. Denote by μ_off_ and $\sigma_{\text{off}}^{2}$ the average and variance of the off-diagonal elements, respectively. We set ${\bar{t}}_{\max}=\max\left(2,\left\lfloor \left(\overset{2}{\underset{\text{on}}{\mu }}-\mu_{\text{off}}^{2}\right)/\sigma_{\text{off}}^{2}\right\rfloor \right)$, where ⌊·⌋ is the largest integer smaller than or equal to the argument. Then, we generate $N_{\text{ROI}}\times {\bar{t}}_{\max}$ variables, denoted by *x*_*i, t*_ (1 ≤ *i* ≤ *N*_ROI_, $1\leq t\leq {\bar{t}}_{\max}$) that independently obey the normal distribution with mean $\sqrt{\mu_{\text{off}}/{\bar{t}}_{\max}}$ and variance $-\mu_{\text{off}}/{\bar{t}}_{\max}+\sqrt{\mu_{\text{off}}^{2}/{\bar{t}}_{\max}^{2}+\sigma_{\text{off}}^{2}/{\bar{t}}_{\max}}$. The H-Q-S algorithm generates a randomized covariance matrix by ${\text{cov}}_{ij}=\overset{{\bar{t}}_{\max}}{\underset{t=1}{\sum }}x_{i\ell }x_{j\ell }$ (1 ≤ *i, j* ≤ *N*_ROI_). In other words, the algorithm assumes that the signal at ROI *i* is a white-noise time series with a positive bias of length ${\bar{t}}_{\max}$, which is independent across the time and ROIs.

### 2.7. White-noise signals

To generate another null model of the covariance matrix, we used white-noise signals. For each ROI, we generated a time series of length 200 in which the signal at each time step and ROI independently obeyed the normal distribution with mean 0 and standard deviation 1. Then, we calculated the covariance matrix using pairs of the *N*_ROI_ time series and converted it into the correlation matrix.

### 2.8. Participants

One-hundred thirty eight (*n* = 138) healthy and right-handed participants (54 females and 84 males) were selected from the Nathan Kline Institute's (NKI) Rockland Sample (Nooner et al., 2012). The NKI's data that we used are publicly available. The age of the participants ranged between 18 and 85 years (mean = 41.7, std = 18.4).

For four of our participants, the H-Q-S algorithm did not work because the average off-diagonal element for the empirical covariance matrix was negative, violating the precondition for the algorithm (Hirschberger et al., 2007). Therefore, we removed the four participants in the analysis that used the H-Q-S algorithm.

### 2.9. fMRI data acquisition and preprocessing

The MRI data were recorded in a 3T scanner (MAGNETOM, TrioTim syngo MR B15, Siemens). fMRI data were obtained during rest with an echo planner imaging (EPI) sequence (TR = 2500 ms, TE = 30 ms, flip angle = 80°, 38 slices, spatial resolution = 3 × 3 × 3 mm^3^, FOV = 216 ms, acquisition time = 10 m 55 s). A total of *t*_max_ = 258 volumes was recorded from each participant. Anatomical images were acquired with T1-weighted sequence (MPRAGE) (TR = 2,500 ms, TE = 3.5 ms, flip angle = 8°, spatial resolution = 1 × 1 × 1 mm^3^). During the EPI data acquisition, the participants were asked to be relaxed with their eyes open.

Data preprocessing was performed using FMRIB's Software Library (FSL; www.fmrib.ox.ac.uk/fsl), including skull stripping of structural images with BET and registration with FLIRT; each functional image was registered to the participant's high-resolution brain-extracted structural image and the standard Montreal Neurological Institute (MNI) 2-mm brain. Functional data were then preprocessed with motion correction with MCFLIRT and smoothing with full-width half-maximum 5 mm. We also applied additional preprocessing steps to the functional data to remove spurious variance. First, we regressed out six head motion parameters, the global signal, cerebrospinal fluid (CSF) signal, and white matter (WM) signal with FSL FEAT. For each participant, CSF, gray matter (GM) and WM were segmented through FSL's FAST based on his/her T1. The signal averaged over all voxels in GM, WM and CSF was used as global signal. We then applied band-pass temporal filtering (0.01–0.1 Hz).

### 2.10. Linear mixed model

To estimate the linear mixed model with a fixed effect and random effects, we used the *lmer* function in lme4 package in R (v.3.4.1). The dependent variable in the linear mixed model was the local clustering coefficient. The fixed and random effects were the node strength and the participant, respectively. To obtain the *P* value, we used the *F*-test with Kenward-Roger approximation implemented as the *KRmodcomp* function in pbkrtest package in R.

## 3. Results

We demonstrate the utility of the proposed clustering coefficients on fMRI data collected from participants of a wide range of the age. We looked for associations of the clustering coefficients with the age and its dependence on the ROIs.

### 3.1. Comparison with null models

Statistically larger values of conventional clustering coefficients have repeatedly been observed in empirical brain networks as compared to the null models (Bullmore and Sporns, 2009; Bassett and Sporns, 2017). Motivated by these studies, we examined whether the amount of clustering was different between the empirical data and these null models after we controlled for the amount of correlation between two ROIs *j* and ℓ expected from an indirect path between *j* and ℓ through a third ROI *i*. For each participant, we compared the proposed clustering coefficients between the fMRI data obtained from all the participants, those calculated for the H-Q-S null model (Hirschberger et al., 2007; Zalesky et al., 2012), and white-noise signals.

The empirical correlation matrices yielded significantly larger values of the clustering coefficient than the correlation matrices for white-noise signals did. The results were consistent between the two definitions of the clustering coefficient, i.e., *C*^cor,A^ [empirical: 0.221 ± 0.029, white noise: 0.057 ± 0.002, *t*_(137)_ = 66.0, *P* < 10^−6^, *d* = 11.28] and *C*^cor,M^ [empirical: 0.031 ± 0.008, white noise: 0.002 ± 0.000, *t*_(137)_ = 40.3, *P* < 10^−6^, *d* = 6.89]. This result is consistent with the previous findings with the conventional clustering coefficients for networks, where empirical functional networks tended to have large clustering coefficients than randomized networks (Eguíluz et al., 2005; Salvador et al., 2005; Achard et al., 2006; Bassett and Bullmore, 2006).

In contrast, the two types of clustering coefficient were smaller for the empirical data than for the randomized data generated by the H-Q-S algorithm [for *C*^cor,A^, H-Q-S: 0.281 ± 0.073, *t*_(133)_ = −12.4, *P* < 10^−6^, *d* = −2.15; for *C*^cor,M^, H-Q-S: 0.056 ± 0.039, *t*_(133)_ = −8.59, *P* < 10^−6^, *d* = −1.49]. This result has probably arisen because the H-Q-S algorithm generates a correlation matrix from short white-noise time series assumed at each ROI. Then, the partial correlation (Equation 5) calculated for the H-Q-S algorithm is distributed relatively widely due to statistical fluctuations, whose distribution can be even wider than that for the empirical data. This fact makes *C*^cor,A^ and *C*^cor,M^, which more or less depends on the absolute value of the partial correlation, large for the randomized data generated by the H-Q-S algorithm.

### 3.2. Age-related differences in the clustering coefficients tailored to correlation matrices

**Figure 2:**
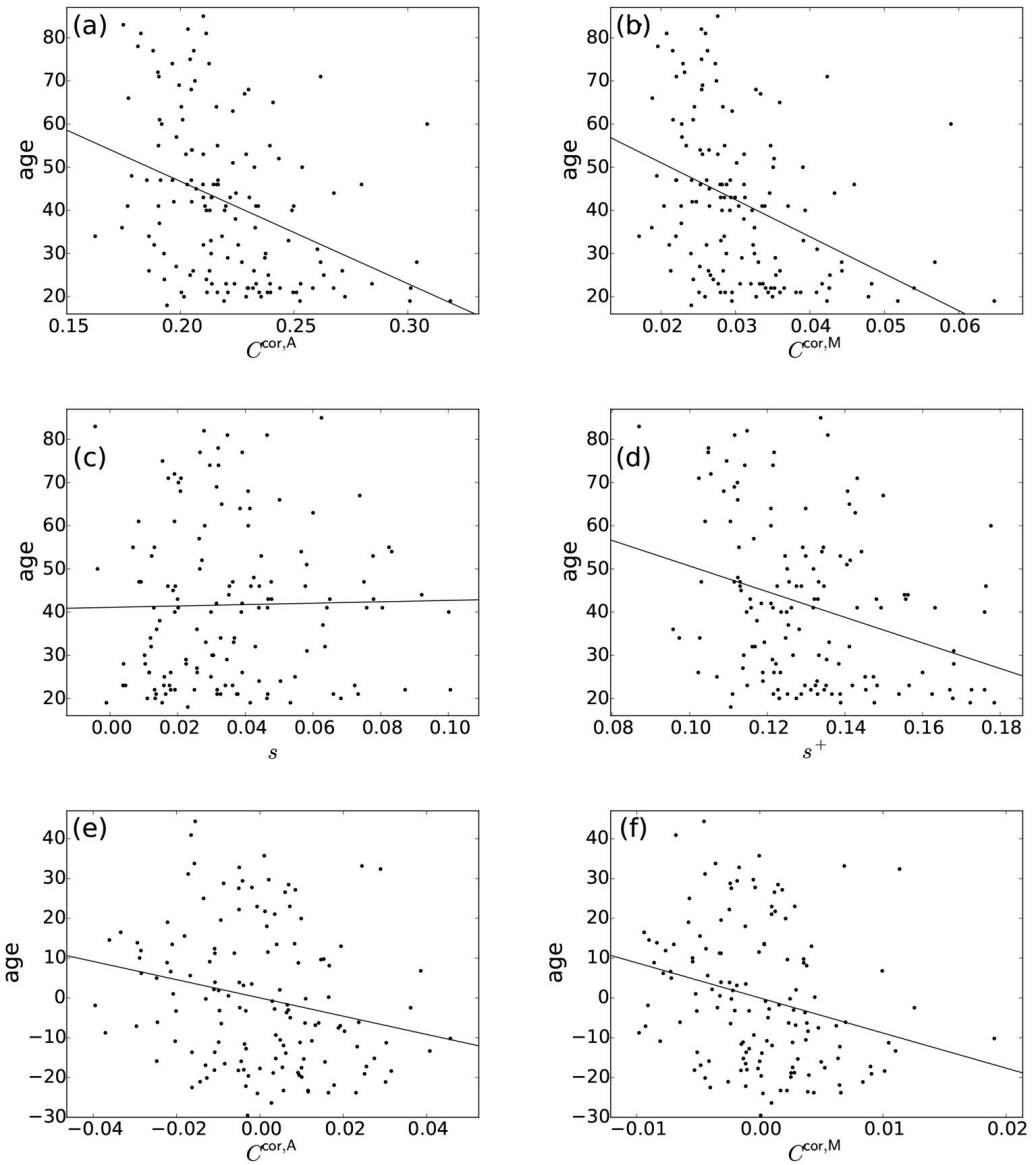
Relationship between the age and network indices. **(a)** *C*^cor,A^ vs. age. **(b)** *C*^cor,M^ vs. age. **(c)** *s* vs. age. **(d)** *s*^+^ vs. age. **(e)** *C*^cor,A^ vs. age, where the effect of *s*^+^ is regressed out. **(f)** *C*^cor,M^ vs. age, where the effect of *s*^+^ is regressed out. A symbol represents an individual. The lines represent the linear fit: **(a)** age = −237.0 × *C*^cor,A^ + 94.1, **(b)** age = −857.5 × *C*^cor,M^ + 68.2, **(c)** age = 16.1 × *s* + 41.1, **(d)** age = −296.8 × *s*^+^ + 80.3, **(e)** age = −229.2 × *C*^cor,A^, **(f)** age = −882.0 × *C*^cor,M^. In **(e,f)**, the linear contribution of *s*^+^ to the variables plotted in **(a,b)** are subtracted from the original variables and the residuals are plotted. The Pearson correlation coefficient between the residuals gives the partial correlation coefficient.

Normal ageing was shown to adversely affect small-worldness of brain networks (Achard and Bullmore, 2007). Because the clustering coefficient is a major index which is used to assess the small-worldness of networks (Watts and Strogatz, 1998), we examined whether our clustering coefficients were able to detect such age-related changes in network structure. We found a negative relationship between each of the two types of clustering coefficients (i.e., *C*^cor,A^ and *C*^cor,M^) and the age [*C*^cor,A^: *r*_(136)_ = −0.377, *P* < 10^−5^; *C*^cor,M^: *r*_(136)_ = −0.397, *P* < 10^−5^; Figures 2a,b, Table 1]. To explore whether the age is correlated with an index that can be more easily calculated than the clustering coefficient, we examined the relationships between the age and two indices of average functional connectivity. We found that the age was uncorrelated with *s* [*r*_(136)_ = 0.020, *P* = 0.82; Figure 2c, Table 1] but negatively correlated with *s*^+^ [*r*_(136)_ = −0.311, *P* = 0.0002; Figure 2d, Table 1]. The two clustering coefficients were also strongly correlated with *s*^+^, whereas they were not correlated with *s* (Table 2). Therefore, we suspected that the negative correlation between the clustering coefficients and the age was caused by the combination of the negative correlation between *s*^+^ and the age and the positive correlation between *s*^+^ and the clustering coefficient. However, significant negative correlation persisted between the clustering coefficients and the age even after controlling for the effect of *s*^+^ [*C*^cor,A^: *r*_(136)_ = −0.224, *P* = 0.0076; *C*^cor,M^: *r*_(136)_ = −0.259, *P* = 0.0019; see Figures 2e,f for the scatter plot between the clustering coefficient and the age after the linear effect of *s*^+^ has been regressed out from both variables; also see Table 1]. This result indicates that the negative correlation between the clustering coefficients and age is not completely explained by *s*^+^. Therefore, *C*^cor,A^ and *C*^cor,M^ quantify effects of the age on fMRI signals beyond what is revealed by the average functional connectivity.

**Table 1:** Correlation between the clustering coefficient and age.

**Index**	**Unconditional**		**Effect of** ***s***^**+**^ **controlled**	
	***r***	***P***	***r***	***P***
**PEARSON CORRELATION MATRIX**				
*C*^cor,A^	−0.377	<10^−5^	−0.224	0.0076
*C*^cor,M^	−0.397	<10^−5^	−0.259	0.0019
*C*^unw^, edge density = 0.1	−0.234	0.0058	−0.104	0.23
*C*^unw^, edge density = 0.2	−0.197	0.021	−0.032	0.71
*C*^wei,B^	−0.262	0.0019	0.018	0.83
*C*^wei,O^	−0.240	0.0045	0.014	0.87
*C*^wei,Z^	−0.229	0.0068	−0.032	0.71
**PARTIAL CORRELATION MATRIX**				
*C*^unw^, edge density = 0.1	−0.001	0.99	0.037	0.67
*C*^unw^, edge density = 0.2	0.048	0.58	0.028	0.75
*C*^wei,B^	−0.056	0.51	−0.022	0.80
*C*^wei,O^	0.057	0.50	0.094	0.27
*C*^wei,Z^	0.057	0.51	0.076	0.37
**AVERAGE CONNECTIVITY**				
*s*	0.020	0.82	–	–
*s*^+^	−0.311	0.0002	–	–

*The correlation coefficient is denoted by r. The degree of freedom is equal to n−2 = 136*.

**Table 2:** Correlation between the clustering coefficient and the node strength.

**Index**	*****s*****		***s***^**+**^	
	***r***	***P***	***r***	***P***
**PEARSON CORRELATION MATRIX**				
*C*^cor,A^	−0.096	0.26	0.812	<10^−15^
*C*^cor,M^	−0.084	0.33	0.798	<10^−15^
*C*^unw^, edge density = 0.1	0.001	0.99	0.471	<10^−8^
*C*^unw^, edge density = 0.2	0.050	0.56	0.550	<10^−11^
*C*^wei,B^	0.359	<10^−4^	0.869	<10^−15^
*C*^wei,O^	0.022	0.80	0.798	<10^−15^
*C*^wei,Z^	−0.080	0.35	0.664	<10^−15^
**PARTIAL CORRELATION MATRIX**				
*C*^unw^, edge density = 0.1	0.021	0.81	0.115	0.18
*C*^unw^, edge density = 0.2	−0.097	0.26	−0.070	0.42
*C*^wei,B^	0.080	0.35	0.113	0.19
*C*^wei,O^	−0.006	0.94	0.100	0.24
*C*^wei,Z^	−0.041	0.64	0.050	0.56

*The degree of freedom is equal to n−2 = 136*.

Positive edges and negative edges may have distinct meanings (Rubinov and Sporns, 2011). Therefore, we examined variants of the proposed clustering coefficients calculated only from positive triangles (denoted by *C*^cor,A,+^ and *C*^cor,M,+^) or negative triangles (denoted by *C*^cor,A,−^ and *C*^cor,M,−^). These variants of clustering coefficients were negatively correlated with the age [*C*^cor,A,+^: *r*_(136)_ = −0.398, *P* < 10^−5^; *C*^cor,A,−^: *r*_(130)_ = −0.291, *P* = 0.0007; *C*^cor,M,+^: *r*_(136)_ = −0.431, *P* < 10^−5^; *C*^cor,M,−^: *r*_(130)_ = −0.304, *P* = 0.0004]. This negative relationship was significant even after controlling for the effect of *s*^+^ [*C*^cor,A,+^: *r*_(136)_ = −0.263, *P* = 0.0019; *C*^cor,A,−^: *r*_(130)_ = −0.197, *P* = 0.024; *C*^cor,M,+^: *r*_(136)_ = −0.315, *P* = 0.0002; *C*^cor,M,−^: *r*_(130)_ = −0.196, *P* = 0.024]. The negative correlation was stronger for the clustering coefficients based on the positive triangles (i.e., *C*^cor,A,+^ and *C*^cor,M,+^) than those based on the negative triangles (i.e., *C*^cor,A,−^ and *C*^cor,M,−^). We conclude that the age-related differences in the clustering coefficients observed with *C*^cor,A^ and *C*^cor,M^ are robust against the restriction of the method to the positive or negative triangles. Note that the age-related decline of *C*^cor,A,+^ and *C*^cor,M,+^ was stronger than that of *C*^cor,A^ and *C*^cor,M^, respectively.

The rationale behind our clustering coefficients is that the correlation between two neighbors of a focal ROI should be discounted due to the effect of the indirect path. The clustering coefficients *C*^cor,A^ and *C*^cor,M^ are not the only indices complying with this rationale. To examine the robustness of our results with respect to specific definitions of the clustering coefficient, we examined the relationship among two other variants of the clustering coefficient designed for correlation matrices and *s*, *s*^+^ and the age. Although the correlation between the clustering coefficient and the age was somewhat weaker than in the case of *C*^cor,A^ and *C*^cor,M^, the results with the other two variants of the clustering coefficient were qualitatively the same as those for *C*^cor,A^ and *C*^cor,M^ (Appendix A).

### 3.3. Age-related differences in the conventional clustering coefficients

We repeated the same analysis using the clustering coefficients previously proposed for unweighted networks (i.e., *C*^unw^) and weighted networks (i.e., *C*^wei,B^, *C*^wei,O^ and *C*^wei,Z^). For unweighted networks, we used two edge density values, 0.1 and 0.2. Qualitatively, the clustering coefficients for unweighted and weighted networks behaved similarly to *C*^cor,A^ and *C*^cor,M^ did. In other words, the clustering coefficients were negatively correlated with the age (Table 1), positively and strongly correlated with *s*^+^ and not with *s* with the exception of *C*^wei,B^ (Table 2). However, the correlation with the age was weaker than in the case of *C*^cor,A^ and *C*^cor,M^ (Table 1; see Appendix B for the statistical results). In fact, the partial correlation between the conventional clustering coefficients (i.e., *C*^unw^, *C*^wei,B^, *C*^wei,O^, and *C*^wei,Z^) and the age was not significant when one controls the effect of *s*^+^ (Table 1). These results suggest that these conventional clustering coefficients extract relatively similar information to that extracted by *s*^+^ as compared to *C*^cor,A^ and *C*^cor,M^ do.

### 3.4. Age-related differences in the clustering coefficients for networks derived from partial correlation matrix

Functional networks are often defined in terms of the partial correlation matrix (Salvador et al., 2005; Marrelec et al., 2006; Smith et al., 2011). Therefore, as a benchmark, we calculated the conventional clustering coefficients (for unweighted and weighted networks) for functional networks defined by the partial correlation matrix. The clustering coefficients were not correlated with *s* or *s*^+^ (Table 2). These clustering coefficients were also uncorrelated with the age (Table 1).

### 3.5. Relationship between the local clustering coefficients and the node strength (weighted degree of the node)

Local clustering coefficients have been used for characterizing individual ROIs (Sporns and Zwi, 2004; Achard et al., 2006; He et al., 2007; Alexander-Bloch et al., 2010; Lynall et al., 2010; Power et al., 2010; van den Heuvel et al., 2010; van den Heuvel and Sporns, 2011; Wee et al., 2011; Fornito et al., 2012; Tijms et al., 2013; Sala-Llonch et al., 2014). In this section we show that, differently from the conventional clustering coefficients, the present clustering coefficients do not confound the strength of local clustering at an ROI and the magnitude of the ROI's connectivity.

**Figure 3:**
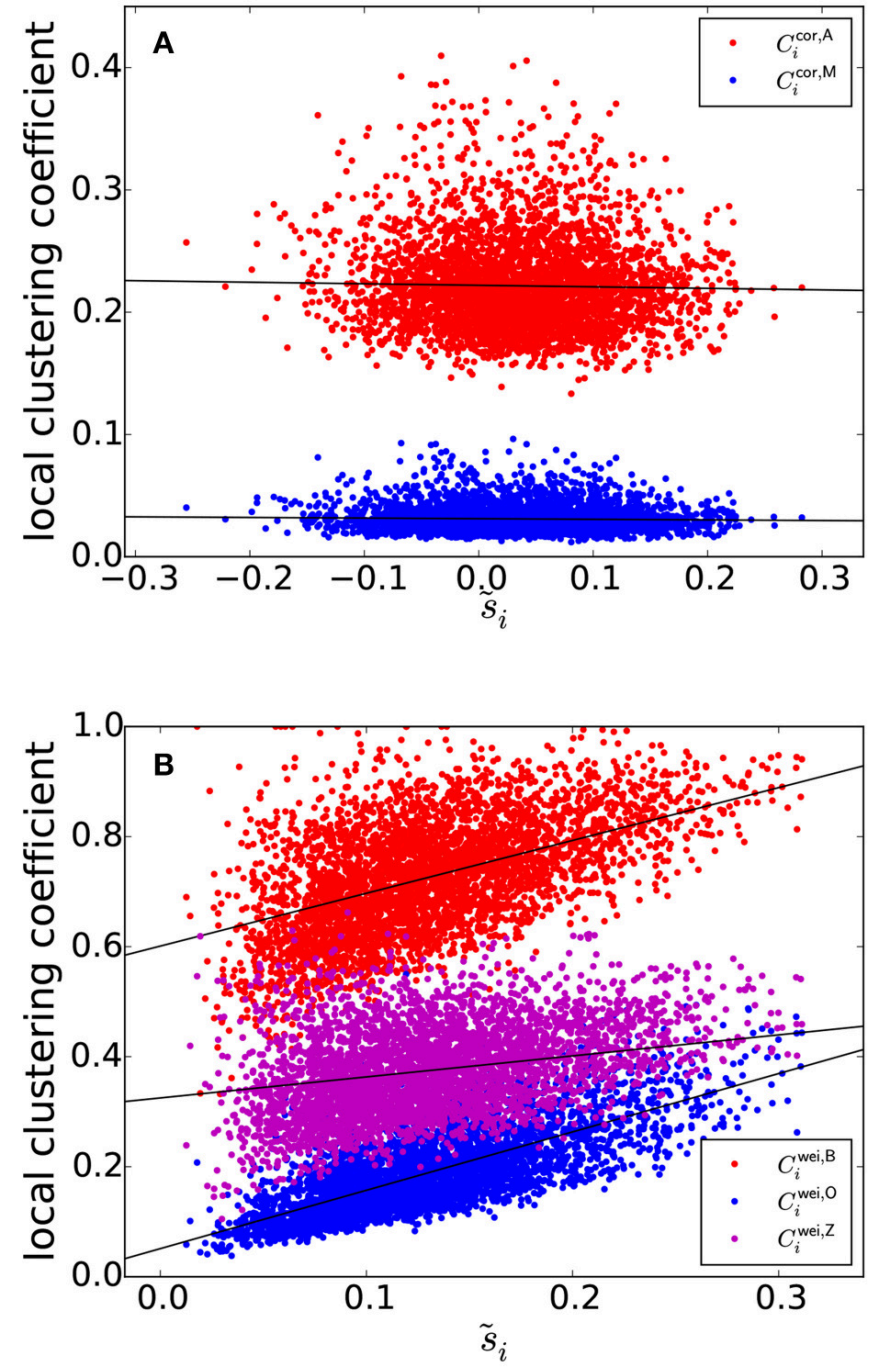
**(A)** Relationship between ${\tilde{s}}_{i}$ and the local clustering coefficients for correlation matrices. **(B)** Relationship between ${\tilde{s}}_{i}$ and the local clustering coefficients for weighted networks. The solid lines represent the fixed effect estimated by the linear mixed model.

The clustering coefficients $C_{i}^{\text{cor},\text{A}}$ and $C_{i}^{\text{cor},\text{M}}$ are plotted against ${\tilde{s}}_{i}\equiv s_{i}/\left(N_{\text{ROI}}-1\right)$, i.e., the node strength normalized between −1 and 1, in Figure 3A, where a symbol represents a combination of an ROI and an individual. Figure 3A suggests that *s*_*i*_ and the local clustering coefficient are uncorrelated. To statistically prove this casual observation, we fitted a linear mixed-effects model for each type of local clustering coefficient. In the linear mixed-effects model, the local clustering coefficient value for the combination of a participant and an ROI was the dependent variable (*n* = 138 participants and *N*_ROI_ = 30 ROIs). The independent variable was the equivalent of the node strength, i.e., $\overset{N_{\text{ROI}}}{\underset{j=1;j\neq i}{\sum }}\rho \left(i,j\right)$. We assumed random effects over participants influencing the slope and intercept. We found that $C_{i}^{\text{cor},\text{A}}$ and $C_{i}^{\text{cor},\text{M}}$ did not show strong positive correlation with $\overset{N_{\text{ROI}}}{\underset{j=1;j\neq i}{\sum }}\rho \left(i,j\right)$ [$C_{i}^{\text{cor},\text{A}}$: *t*_(4139)_ = −2.33, *P* = 0.023, Pearson correlation coefficient between $C_{i}^{\text{cor},\text{A}}$ and $\overset{N_{\text{ROI}}}{\underset{j=1;j\neq i}{\sum }}\rho \left(i,j\right)$, *i* = 1, …, *N*_ROI_ for each participant, which is then averaged over all the participants, as a measure of effect size *r*_(28)_ = −0.023, $C_{i}^{\text{cor},\text{A}}=-0.013{\tilde{s}}_{i}+0.222$; $C_{i}^{\text{cor},\text{M}}$: *t*_(4139)_ = −3.20, *P* = 0.0019, *r*_(28)_ = −0.047, $C_{i}^{\text{cor},\text{M}}=-0.0050{\tilde{s}}_{i}+0.031$]. Note that the effect size as measured by *r*_(28)_ was small, although the effects were significant owing to a large sample size.

We investigated the same linear relationship for the correlation matrices generated by the randomization of the original correlation matrices using the H-Q-S algorithm. We generated one null model network per participant. For four participants, the algorithm did not work because the average off-diagonal element of the covariance matrix for the empirical covariance matrix was negative, violating the condition for the algorithm to be used (Hirschberger et al., 2007). For the remaining *n*−4 = 134 participants, the dependence of the local clustering coefficient of ROI *i* on $\overset{N_{\text{ROI}}}{\underset{j=1;j\neq i}{\sum }}\rho \left(i,j\right)$ remained small [$C_{i}^{\text{cor},\text{A}}$: *t*_(4019)_ = −1.93, *P* = 0.059, *r*_(28)_ = −0.021, $C_{i}^{\text{cor},\text{A}}=-0.0051{\tilde{s}}_{i}+0.28$; $C_{i}^{\text{cor},\text{M}}$: *t*_(4019)_ = −1.21, *P* = 0.23, *r*_(28)_ = −0.019, $C_{i}^{\text{cor},\text{M}}=-0.0016{\tilde{s}}_{i}+0.055$]. Therefore, we conclude that $C_{i}^{\text{cor},\text{A}}$ and $C_{i}^{\text{cor},\text{M}}$ (and hence *C*^cor,A^ and *C*^cor,M^) are not affected by pseudo-correlation and provide measurements orthogonal to the node strength.

In contrast, the previously provided local clustering coefficients for unweighted or weighted networks [i.e., $C_{i}^{\text{unw}}$, $C_{i}^{\text{wei},\text{B}}$, $C_{i}^{\text{wei},\text{O}}$, and $C_{i}^{\text{wei},\text{Z}}$ given by Equations (1), (2), (3), and (4), respectively] should be correlated with the degree (i.e., the number of edges connected to a node), *k*_*i*_ (in the case of unweighted networks) or node strength, i.e., weighted degree *s*_*i*_ (in the case of weighted networks) when applied to correlation matrices. Let us explain this point for weighted networks for the sake of clarity. Because of indirect paths, if *w*_*ij*_ and *w*_*iℓ*_ are large, *w*_*jℓ*_ tends to be large, which increases the value of the local clustering coefficient of ROI *i*. At the same time, *s*_*i*_ is large if *w*_*ij*_ and *w*_*iℓ*_ are. Therefore, we expect systematic positive correlation between *s*_*i*_ and any of $C_{i}^{\text{unw}}$, $C_{i}^{\text{wei},\text{B}}$, $C_{i}^{\text{wei},\text{O}}$, and $C_{i}^{\text{wei},\text{Z}}$.

The three types of clustering coefficient for weighted networks ($C_{i}^{\text{wei},\text{B}}$, $C_{i}^{\text{wei},\text{O}}$, and $C_{i}^{\text{wei},\text{Z}}$) are plotted against ${\tilde{s}}_{i}$ in Figure 3B. We did not examine the local clustering coefficient for unweighted networks (i.e., $C_{i}^{\text{unw}}$) because it was undefined for many ROIs, whose nodal degree *k*_*i*_ was either 0 or 1; our network is relatively small (i.e., *N*_ROI_ = 30) and the edge density is not assumed to be too large. The three local weighted clustering coefficients and ${\tilde{s}}_{i}$ were strongly correlated [$C_{i}^{\text{wei},\text{B}}$: *t*_(4139)_ = 23.7 for the fixed effects of ${\tilde{s}}_{i}$, *P* < 10^−15^, *r*_(28)_ = 0.43, the estimated fixed effects: $C_{i}^{\text{wei},\text{B}}=0.960{\tilde{s}}_{i}+0.601$; $C_{i}^{\text{wei},\text{O}}$: *t*_(4139)_ = 43.4, *P* < 10^−15^, *r*_(28)_ = 0.70, $C_{i}^{\text{wei},\text{O}}=0.950{\tilde{s}}_{i}+0.064$; $C_{i}^{\text{wei},\text{Z}}$: *t*_(4139)_ = 10.8, *P* < 10^−15^, *r*_(28)_ = 0.27, $C_{i}^{\text{wei},\text{Z}}=0.382{\tilde{s}}_{i}+0.325$].

Upon randomization of the original correlation matrices by the H-Q-S algorithm, the positive relationship between the local clustering coefficient and ${\tilde{s}}_{i}$ persisted for each weighted clustering coefficient index [$C_{i}^{\text{wei},\text{B}}$: *t*_(4019)_ = 13.1, *P* < 10^−15^, *r*_(28)_ = 0.27, $C_{i}^{\text{wei},\text{B}}=0.509{\tilde{s}}_{i}+0.595$; $C_{i}^{\text{wei},\text{O}}$: *t*_(4019)_ = 37.0, *P* < 10^−15^, *r*_(28)_ = 0.60, $C_{i}^{\text{wei},\text{O}}=0.628{\tilde{s}}_{i}+0.100$; $C_{i}^{\text{wei},\text{Z}}$: *t*_(4019)_ = 8.56, *P* = 3.7 × 10^−13^, *r*_(28)_ = 0.17, $C_{i}^{\text{wei},\text{Z}}=0.217{\tilde{s}}_{i}+0.355$]. These results suggest that these local clustering coefficients are confounded by the effect of node strength, which could arise from the pseudo-correlation due to indirect paths.

### 3.6. Dependence of the local clustering coefficients on the brain system

Previous studies found systematic regional differences (e.g., across different lobes) in the local clustering coefficient in functional brain networks (Achard et al., 2006; Alexander-Bloch et al., 2010; Lynall et al., 2010; Sala-Llonch et al., 2014). However, this effect may be confounded by the effect of the node strength. As a case study, in this section we show that we do not see the association between previously defined brain systems (i.e., subsets of the ROIs constituting the entire network) and age-related changes in conventional local clustering coefficients if the effect of the node strength is controlled.

We first calculated the Pearson correlation coefficient (*r*) between the age and a nodal index such as the local clustering coefficient at each ROI. Then, we examined whether *r* was different across three brain systems whose functions and structures have been examined (Fair et al., 2009; Power et al., 2011): the default mode network (DMN), cingulo-opercular network (CON) and fronto-parietal network (FPN).

**Figure 4:**
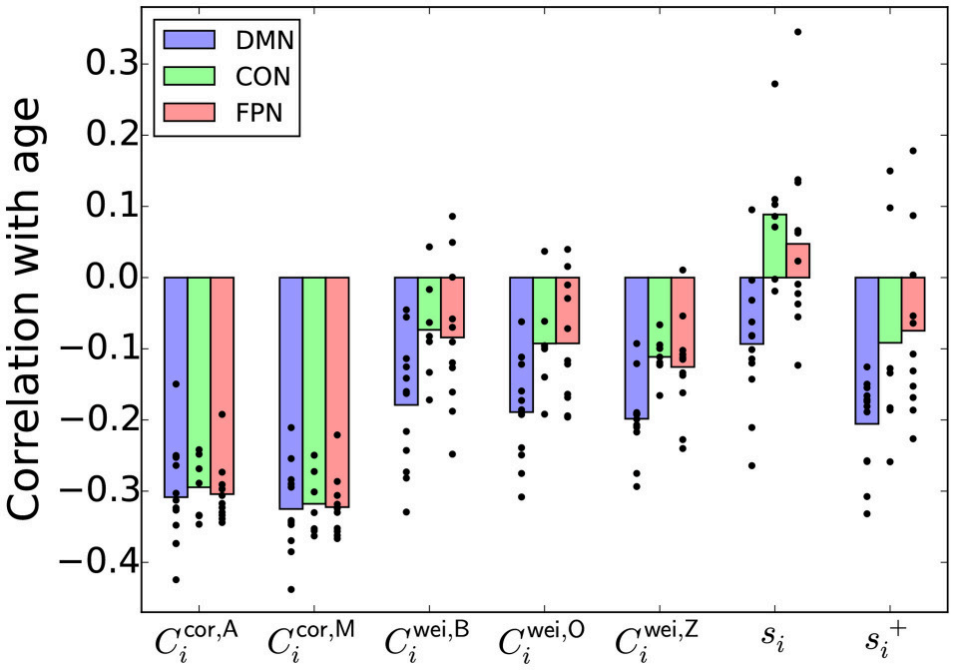
Pearson correlation coefficient between a nodal index and the age, averaged over the ROIs in the DMN, CON, or FPN. The circle represents the correlation coefficient value for a single node.

The *r* values between various nodal indices and the age, averaged over the ROIs in each of the DMN, CON, and FPN, are shown in Figure 4. For the clustering coefficients for weighted networks (i.e., *C*^wei,B^, *C*^wei,O^, and *C*^wei,Z^), *r* was negative for most ROIs, confirming the results reported in section 3.2 that the (global) clustering coefficient was negatively correlated with the age of the participant. The *r* value was different between the three brain systems for each type of weighted clustering coefficient [$C_{i}^{\text{wei},\text{B}}$: *F*_(2, 27)_ = 4.32, *P* = 0.023, η^2^ = 0.24; $C_{i}^{\text{wei},\text{O}}$: *F*_(2, 27)_ = 5.69, *P* = 0.0087, η^2^ = 0.30; *C*^wei,Z^: *F*_(2, 27)_ = 6.87, *P* = 0.0039, η^2^ = 0.34; a one-way factorial analysis of variance (ANOVA) [System: DMN/CON/FPN]]. *Post-hoc* two-sample *t*-tests revealed that the effect of the age was larger in the DMN than in the CON and FPN [$C_{i}^{\text{wei},\text{B}}$, DMN − CON: *t*_(17)_ = −2.64, *P* = 0.017, *d* = −1.28; $C_{i}^{\text{wei},\text{B}}$, DMN − FPN: *t*_(21)_ = −2.38, *P* = 0.017, *d* = −1.04; $C_{i}^{\text{wei},\text{O}}$, DMN − CON: *t*_(17)_ = −2.86, *P* = 0.011, *d* = −1.39; $C_{i}^{\text{wei},\text{O}}$, DMN − FPN: *t*_(21)_ = −2.95, *P* = 0.00077, *d* = −1.29; $C_{i}^{\text{wei},\text{Z}}$, DMN − CON: *t*_(17)_ = −3.84, *P* = 0.0013, *d* = −1.86; $C_{i}^{\text{wei},\text{Z}}$, DMN − FPN: *t*_(21)_ = −2.78, *P* = 0.011, *d* = −1.21].

However, qualitatively the same association between the age and the brain system was also found when *r* was defined as the correlation between the node strength (i.e., *s*_*i*_) and the age [*F*_(2, 27)_ = 8.01, *P* = 0.0019, η^2^ = 0.37] and when *r* was defined as the correlation between $s_{i}^{+}$, which was defined as $\overset{N_{\text{ROI}}}{\underset{j=1;\rho \left(i,j\right)>0}{\sum }}\rho \left(i,j\right)$, and the age [*F*_(2, 27)_ = 4.43, *P* = 0.022, η^2^ = 0.25]. Because the local clustering coefficients for weighted networks (i.e., $C_{i}^{\text{wei},\text{B}}$, $C_{i}^{\text{wei},\text{O}}$, and $C_{i}^{\text{wei},\text{Z}}$) were positively correlated with the node strength and $s_{i}^{+}$, we take *s*_*i*_ or $s_{i}^{+}$ as a simpler signature of the system dependence of the age effect than the local clustering coefficient.

In contrast, the proposed local clustering coefficients, which were not correlated with *s*_*i*_ or $s_{i}^{+}$ (Figure 3A), were not different across the brain systems [$C_{i}^{\text{cor},\text{A}}$: *F*_(2, 27)_ = 0.13, *P* = 0.88, η^2^ = 0.01; $C_{i}^{\text{cor},\text{M}}$: *F*_(2, 27)_ = 0.04, *P* = 0.96, η^2^ = 0.003; also see Figure 4]. These observations suggest that the apparent dependence of the clustering coefficient on the brain system when a conventional clustering coefficient is used is explained by the nodal measure, *s*_*i*_ or $s_{i}^{+}$.

We found similar results in sensory-motor regions in the brain (Appendix C). In other words, the association between the clustering coefficient and the age is more positive for the ROIs in a somatosensory-motor system than for the ROIs in an auditory system and a visual system when we used the clustering coefficients for weighted networks. Qualitatively the same dependence on the brain system was also found when we looked at the association between the node strength and the age. In contrast, with the proposed local clustering coefficients, the auditory system showed the strongest association between the clustering coefficient and the age. These results bear robustness to our suggestion that the proposed local clustering coefficients are not confounded by the node's strength, whereas the conventional clustering coefficients are.

## 4. Discussion

We proposed two clustering coefficients tailored to correlation matrices. They do not suffer from pseudo-correlation induced by indirect paths between two ROIs through a third ROI, do not require thresholding, do not discard negative pairwise correlation, and do not suffer from the difficulty in estimating partial correlation matrices. The proposed clustering coefficients were more strongly correlated with the participants' age than the conventional clustering coefficients, including those calculated for partial correlation matrices, were. In addition, our clustering coefficients can be used as a local measure to characterize nodes, whereas the counterparts for the conventional clustering coefficients were confounded with the (weighted) degree of the node. These results hold true for two alternative definitions of the clustering coefficient for correlation matrices that we additionally propose (Appendix A).

Previous research has produced incongruent results regarding the changes in the clustering coefficient along ageing. In an fMRI study, both at rest and during tasks, the clustering coefficient in functional networks decreased with ageing (Grady et al., 2016), which is consistent with the present results. This observation is also consistent with results of an EEG study at rest (Knyazev et al., 2015). In different studies, however, no difference was found in the clustering coefficient between younger and older individuals (Wang et al., 2010; Brier et al., 2014), or the clustering coefficient increased with ageing (Matthäus et al., 2012; Zhu et al., 2012; Liu et al., 2014; Sala-Llonch et al., 2014). The diversity in these results may owe to participant's heterogeneity, inefficiency of the conventional clustering coefficients or other reasons. It should be noted that the decrease in the clustering coefficient found in a recent study (Grady et al., 2016) and the present study is consistent with the decline in small-worldness of brain networks, which have been documented by using different indices (Achard and Bullmore, 2007; Gong et al., 2009). However, we do not claim that the decline in the clustering coefficient along ageing is a ground truth. In fact, the coordinates of the ROIs in the current data set were determined from participants aged 7–31 (Fair et al., 2009) so that they may not reflect functional ROIs in older adults (Chan et al., 2014; Geerligs et al., 2017). This issue warrants further study.

We demonstrated the utility of the proposed correlation coefficients with fMRI data collected from individuals of different ages. They may also be useful in deciphering functional brain networks collected from different types of individuals such as those with psychiatric or other disorders, those under different task conditions and children under developments. Furthermore, the present method can be used to any correlation or covariance matrix, thus promising their applicability to other functional data of the brain, such as EEG, MEG, correlation in the cortical thickness between ROIs, where correlation is calculated across individuals (see Introduction for references), and even correlation data outside neuroscience.

The proposed clustering coefficients are expected to find immediate applications in the assessment of small-worldness. In the small-world analysis, a major method is to combine the clustering coefficient and the average path length between a pair of nodes, denoted by *L*. When *L* is small and the clustering coefficient is large, one says that the network is small-world (Watts and Strogatz, 1998; Bullmore and Sporns, 2009) (but see Achard and Bullmore, 2007; Gong et al., 2009 for different definitions based on the so-called network efficiency indices). In neuroscience, it is often the case to combine these two indices to examine a single small-worldness index (Humphries et al., 2006) (also see Muldoon et al., 2016 for a recent development). The motivation behind the present study is that the definition or measurement of clustering is nontrivial for correlation matrices, i.e., functional data.

The same caution applies to the path length. A common way to calculate the path length in correlation data is to threshold on the correlation matrix to generate an unweighted network and then measure the path length. However, this method suffers from arbitrariness of thresholding, as discussed in Introduction. Another common way is to define a relationship between the edge weight, i.e., correlation coefficient value, and the cost of passing through the edge. Popular choices of the cost function are the reciprocal of the edge weight (Rubinov and Sporns, 2010) and a constant subtracted by the edge weight (Achard and Bullmore, 2007; Gong et al., 2009). However, the theoretical basis of these decisions seems unclear. A more sensible definition of the distance between ROIs *i* and *j* may be $\sqrt{2\left(1-\rho \left(i,j\right)\right)}$, which qualifies as a Euclidean distance (Mantegna and Stanley, 2000).

We used the three-way partial correlation coefficient controlling for a single ROI to define the clustering coefficients. In contrast, some previous studies derived functional networks from partial correlation matrices (Salvador et al., 2005; Marrelec et al., 2006; Smith et al., 2011). Both types of methods intend to remove the spurious correlation induced by indirect paths between ROIs. While getting common, the methods based on partial correlation matrices face a technical challenge that the partial correlation matrix cannot be determined uniquely from data in general (Schäfer and Strimmer, 2005; Ryali et al., 2012; Brier et al., 2015). In addition, its calculation for a single pair of nodes involves all the other *N*_ROI_ − 2 nodes, contradicting the original premise of the clustering coefficient that it is a local quantity (Watts and Strogatz, 1998). Our clustering coefficients, which use the three-way partial correlation coefficient, do not suffer from the non-uniqueness problem and is a local quantity. Furthermore, we showed that the present clustering coefficients were associated with the age, whereas those calculated for the partial correlation matrices were not. Generalization of this finding to different ROIs, data sets and types of participants, such as those with a particular brain-related disorder, warrants future work.

## Author contributions

NM designed the research. MS preprocessed the data. NM and TE analyzed the data. NM, MS, TE, and TW discussed the results and wrote the manuscript.

### Conflict of interest statement

The authors declare that the research was conducted in the absence of any commercial or financial relationships that could be construed as a potential conflict of interest.
