# Supplementary material for: Clustering Coefficients for Correlation Networks
Source: Front Neuroinform. 2018 Mar 15;12:7. doi: 10.3389/fninf.2018.00007 (PMC5863042; doi:10.3389/fninf.2018.00007)
Supplement: Supplementary file 1 [file DataSheet1.pdf]

Appendix for "Clustering coefficients for correlation networks"

Naoki Masuda, Michiko Sakaki, Takahiro Ezaki, Takamitsu Watanabe

Appendix A. Two alternative clustering coefficients for correlation matrices

We assessed two alternative clustering coefficients for correlation matrices on the empirical and randomised data.

In the first variant, we restricted ourselves to the cases in which $\rho(i,j)$ and $\rho(i,\ell)$ were positive when calculating the local clustering coefficient at ROI i, denoted by $C_i^{\mathrm{cor,P}}$ (superscript P standing for positive). We set

$$C_i^{\mathrm{cor,P}} \equiv \frac{\sum_{\substack{1\le j<\ell\le N_{\mathrm{ROI}} \\ j,\ell\neq i;\rho(i,j),\rho(i,\ell)>0}} \rho(i,j)\rho(i,\ell)\rho^{\mathrm{partial}}(j,\ell \mid i)}{\sum_{\substack{1\le j<\ell\le N_{\mathrm{ROI}} \\ j,\ell\neq i;\rho(i,j),\rho(i,\ell)>0}} \rho(i,j)\rho(i,\ell)}. \tag{A.1}$$

In other words, $C_i^{\mathrm{cor,P}}$ is a weighted average of the partial correlation over pairs of j and ℓ ($j,\ell \neq i, j\neq \ell$) for which $\rho(i,j),\rho(i,\ell)>0$. The corresponding global clustering coefficient, denoted by $C^{\mathrm{cor,P}}$, is given by the average of $C_i^{\mathrm{cor,P}}$ over all nodes. Note that $-1 \le C_i^{\mathrm{cor,P}} \le 1$ ($1\le i\le N_{\mathrm{ROI}}$) and that $-1\le C^{\mathrm{cor,P}}\le 1$.

The second variant of the clustering coefficient uses contributions of all ROIs regardless of the sign of $\rho(i,j)$ and $\rho(i,\ell)$, but in a different manner from $C_i^{\mathrm{cor,A}}$ (and hence $C^{\mathrm{cor,A}}$). If $\rho(i,j),\rho(i,\ell)<0$, Eq. (5) implies that $\rho(j,\ell)$ would be positive if there is no partial correlation between j and ℓ. Therefore, we regard that $\rho^{\mathrm{partial}}(j,\ell \mid i)$ measures the excess correlation between j and ℓ as usual. In contrast, if $\rho(i,j)\rho(i,\ell)<0$, Eq. (5) implies that $\rho(j,\ell)$ would be negative if there is no partial correlation between j and ℓ. This observation is consistent with Heider's balance theory, originating from social psychology and respected in various signed network data, which dictates that in signed unweighted networks (edge weight is either $+1$ or -1), triangles with one or three $+1$'s are stable, whereas those with zero or two $+1$'s are unstable [1–4]. A related remark was previously made for correlation matrices [5]. When $\rho(i,j)\rho(i,\ell)<0$, we regard that $\rho(j,\ell)$ being more negative (towards -1) than $\rho(i,j)\rho(i,\ell)$ is a signature of strong association between ROIs j and ℓ with the influence of ROI i controlled. In other words, we take a negative large value of $\rho^{\mathrm{partial}}(j,\ell \mid i)$ as an indication of clustering composed of the three ROIs, i, j and ℓ, from the viewpoint of i. On the basis of this reasoning, we define the local clustering coefficient denoted

by $C_i^{\mathrm{cor,H}}$ (superscript H standing for Heider) as follows:

$$C_i^{\mathrm{cor,H}} = \frac{\sum_{\substack{1\le j<\ell\le N_{\mathrm{ROI}}\\ j,\ell\neq i}} \rho(i,j)\rho(i,\ell)\rho^{\mathrm{partial}}(j,\ell \mid i)}{\sum_{\substack{1\le j<\ell\le N_{\mathrm{ROI}}\\ j,\ell\neq i}} |\rho(i,j)\rho(i,\ell)|}. \tag{A.2}$$

The denominator on the right-hand side of Eq. (A.2) is for normalisation to guarantee $-1 \le C_i^{\mathrm{cor,H}} \le 1$ ($1 \le i \le N$). The corresponding global clustering coefficient, denoted by $C^{\mathrm{cor,H}}$, is given by the average of $C_i^{\mathrm{cor,H}}$ over all nodes. Note that $-1 \le C^{\mathrm{cor,H}} \le 1$. We also note that $C_i^{\mathrm{cor,P}}$, $C^{\mathrm{cor,P}}$, $C_i^{\mathrm{cor,H}}$ and $C^{\mathrm{cor,H}}$ can be negative, which is different from the clustering coefficients for unweighted and weighted networks and also from $C_i^{\mathrm{cor,A}}$, $C^{\mathrm{cor,A}}$, $C_i^{\mathrm{cor,M}}$ and $C^{\mathrm{cor,M}}$.

Both $C^{\mathrm{cor,P}}$ and $C^{\mathrm{cor,H}}$ were larger for the empirical data than for white-noise signals (for $C^{\mathrm{cor,P}}$, empirical: mean $\pm$ sd $= 0.109 \pm 0.038$, white noise: 0.0003 ± 0.0044, $t_{137} = 33.4$, $P < 10^{-6}$, $d = 5.71$; for $C^{\mathrm{cor,H}}$, empirical: 0.090 ± 0.040, white noise: -0.0001 ± 0.0017, $t_{137} = 26.7$, $P < 10^{-6}$, $d = 4.57$). This result is consistent with that for $C^{\mathrm{cor,A}}$ and $C^{\mathrm{cor,M}}$. In addition, the empirical $C^{\mathrm{cor,P}}$ and $C^{\mathrm{cor,H}}$ values were larger than those for randomised signals generated by the H-Q-S algorithm (for $C^{\mathrm{cor,P}}$, H-Q-S: mean $\pm$ sd $= 0.035 \pm 0.032$, $t_{133} = 22.0$, $P < 10^{-6}$, $d = 3.81$; for $C^{\mathrm{cor,H}}$, H-Q-S: 0.003 ± 0.017, $t_{133} = 25.8$, $P < 10^{-6}$, $d = 4.47$). This result is opposite to that for $C^{\mathrm{cor,A}}$ and $C^{\mathrm{cor,M}}$. In sum, $C^{\mathrm{cor,P}}$ and $C^{\mathrm{cor,H}}$ were larger for the empirical data than for both types of randomised data.

Similar to $C^{\mathrm{cor,A}}$ and $C^{\mathrm{cor,M}}$, we found that $C^{\mathrm{cor,P}}$ and $C^{\mathrm{cor,H}}$ were strongly correlated with s^+ ($C^{\mathrm{cor,P}}$: $r_{136} = 0.874$, $P < 10^{-6}$; $C^{\mathrm{cor,H}}$: $r_{136} = 0.773$, $P < 10^{-6}$) but not with s ($C^{\mathrm{cor,P}}$: $r_{136} = 0.197$, $P = 0.020$; $C^{\mathrm{cor,H}}$: $r_{136} = -0.104$, $P = 0.23$). The correlation between these clustering coefficients and the age was moderate ($C^{\mathrm{cor,P}}$: $r_{136} = -0.310$, $P = 2.1 \times 10^{-4}$; $C^{\mathrm{cor,H}}$: $r_{136} = -0.329$, $P < 8.3 \times 10^{-5}$) but was not significant when we control for the effect of s^+ ($C^{\mathrm{cor,P}}$: $r_{136} = -0.083$, $P = 0.33$; $C^{\mathrm{cor,H}}$: $r_{136} = -0.146$, $P = 0.087$).

To conclude, the results regarding the association of $C^{\mathrm{cor,P}}$ and $C^{\mathrm{cor,H}}$ with the age are consistent with but weaker than those for $C^{\mathrm{cor,A}}$ and $C^{\mathrm{cor,M}}$.

Local clustering coefficients $C_i^{\mathrm{cor,P}}$ and $C_i^{\mathrm{cor,H}}$ did not show strong positive correlation with the equivalent of the node strength, i.e., $\sum_{j=1;j\neq i}^{N_{\mathrm{ROI}}} \rho(i,j)$ ($C_i^{\mathrm{cor,P}}$: $t_{4139} = -1.58$, $P = 0.12$, $r_{28} = -0.057$, $C_i^{\mathrm{cor,P}} = -0.043\tilde{s}_i + 0.110$; $C_i^{\mathrm{cor,H}}$: $t_{4139} = -2.86$, $P = 0.0053$, $r_{28} = -0.075$, $C_i^{\mathrm{cor,H}} = -0.033\tilde{s}_i + 0.091$). Note that the Pearson correlation coefficient values were small, whereas the t value was significant due to a large sample size. Upon randomisation, the dependence of the local clustering coefficient of ROI i on $\sum_{j=1;j\neq i}^{N_{\mathrm{ROI}}} \rho(i,j)$ remained small in terms of the r value ($C_i^{\mathrm{cor,P}}$: $t_{4019} = -5.14$, $P = 1.0 \times 10^{-6}$, $r_{28} = -0.19$, $C_i^{\mathrm{cor,P}} = -0.110\tilde{s}_i + 0.038$; $C_i^{\mathrm{cor,H}}$: $t_{4019} = 0.746$, $P = 0.46$, $r_{28} = 0.011$, $C_i^{\mathrm{cor,H}} = 0.005\tilde{s}_i + 0.000$). Therefore, we conclude that $C_i^{\mathrm{cor,P}}$ and $C_i^{\mathrm{cor,H}}$, and hence $C^{\mathrm{cor,P}}$ and $C^{\mathrm{cor,H}}$ also, are not affected by indirect correlation and provide measurements orthogonal to the node strength.

Appendix B. Difference between the proposed and conventional clustering coefficients in terms of their association with the age

Table 1 suggests that the correlation between the proposed clustering coefficients and the age is stronger than that between the conventional clustering coefficients and the age. To examine this point statistically, we ran the Williams' t-test for two non-independent correlation coefficients with a variable in common [6]. The common variable was the age. We compared each of the two proposed clustering coefficients, $C^{\mathrm{cor,A}}$ and $C^{\mathrm{cor,M}}$, with each of the five conventional clustering coefficients, C^{unw} with edge density 0.1, C^{unw} with edge density 0.2, $C^{\mathrm{wei,B}}$, $C^{\mathrm{wei,O}}$ and $C^{\mathrm{wei,Z}}$. The results ($C^{\mathrm{cor,A}}$ vs C^{unw} with edge density 0.1: $t_{135} = -1.93$, $p = 0.028$; $C^{\mathrm{cor,A}}$ vs C^{unw} with edge density 0.2: $t_{135} = -2.58$, $p = 0.0055$; $C^{\mathrm{cor,A}}$ vs $C^{\mathrm{wei,B}}$: $t_{135} = -1.86$, $p = 0.033$; $C^{\mathrm{cor,A}}$ vs $C^{\mathrm{wei,O}}$: $t_{135} = -3.09$, $p = 0.0012$; $C^{\mathrm{cor,A}}$ vs $C^{\mathrm{wei,Z}}$: $t_{135} = -3.03$, $p = 0.0015$; $C^{\mathrm{cor,M}}$ vs C^{unw} with edge density 0.1: $t_{135} = -2.26$, $p = 0.013$; $C^{\mathrm{cor,M}}$ vs C^{unw} with edge density 0.2: $t_{135} = -2.89$, $p = 0.0022$; $C^{\mathrm{cor,M}}$ vs $C^{\mathrm{wei,B}}$: $t_{135} = -2.14$, $p = 0.017$; $C^{\mathrm{cor,M}}$ vs $C^{\mathrm{wei,O}}$: $t_{135} = -3.10$, $p = 0.0012$; $C^{\mathrm{cor,M}}$ vs $C^{\mathrm{wei,Z}}$: $t_{135} = -3.08$, $p = 0.0013$; all p values were not corrected for multiple comparison) indicate that seven out of the ten cases survived Bonferroni correction ($\alpha = 5\%$). Therefore, we conclude that the two proposed clustering coefficients are more strongly associated with the age than the conventional clustering coefficients are.

Appendix C. Dependence of the local clustering coefficients on sensory-motor brain systems

We repeated the same analysis as that in section 3.6 for sensory-motor brain systems. Because the brain atlas used in the main text only has the DMN, CON, FPN and cerebellum [7], we used the somatosensory-motor network (SMN; 34 ROIs), auditory network (13 ROIs) and visual network (31 ROIs), which are among several brain systems identified in a different study [8].

The Pearson correlation coefficient, r, between the different clustering coefficients and the age and that between the node strength and the age, averaged over the ROIs in each of the SMN, auditory network and visual network, is shown in Fig. C.1. For the clustering coefficients for weighted networks (i.e., $C^{\mathrm{wei,B}}$, $C^{\mathrm{wei,O}}$ and $C^{\mathrm{wei,Z}}$), r was slightly positive on average in the SMN and moderately or considerably negative in the auditory and visual networks. The r value was different between the three brain systems for each type of weighted clustering coefficient ($C_i^{\mathrm{wei,B}}$: $F_{2,75} = 72.6$, $P < 10^{-15}$, $\eta^2 = 0.66$; $C_i^{\mathrm{wei,O}}$: $F_{2,75} = 72.0$, $P < 10^{-15}$, $\eta^2 = 0.66$; $C^{\mathrm{wei,Z}}$: $F_{2,75} = 211$, $P < 10^{-15}$, $\eta^2 = 0.85$; a one-way ANOVA [System: SMN/Auditory/Visual]). Post-hoc two-sample t-tests revealed that the effect of the age was more positive in the SMN than in the auditory and visual networks ($C_i^{\mathrm{wei,B}}$, SMN − Auditory: $t_{45} = 6.37$, $P = 8.9 \times 10^{-9}$, $d = 1.90$; $C_i^{\mathrm{wei,B}}$, SMN − Visual: $t_{63} = 12.8$, $P < 10^{-15}$, $d = 3.23$; $C_i^{\mathrm{wei,O}}$, SMN − Auditory: $t_{45} = 6.79$, $P = 2.1 \times 10^{-9}$, $d = 2.03$;

$C_i^{\mathrm{wei,O}}$, SMN − Visual: $t_{63} = 12.3$, $P < 10^{-15}$, $d = 3.10$; $C_i^{\mathrm{wei,Z}}$, SMN − Auditory: $t_{45} = 8.30$, $P = 1.3 \times 10^{-11}$, $d = 2.47$; $C_i^{\mathrm{wei,Z}}$, SMN − Visual: $t_{63} = 21.8$, $P < 10^{-15}$, $d = 5.49$). However, as is observed in Fig. C.1, qualitatively the same association between the age and the brain system was also found when r was defined as the correlation between s_i and the age ($F_{2,75} = 11.6$, $P = 4.2 \times 10^{-5}$, $\eta^2 = 0.92$) and when r was defined as the correlation between s_i^+ and the age ($F_{2,75} = 25.5$, $P = 3.6 \times 10^{-9}$, $\eta^2 = 0.96$).

The proposed local clustering coefficients were also different across the brain systems ($C_i^{\mathrm{cor,A}}$: $F_{2,75} = 9.06$, $P = 0.00030$, $\eta^2 = 0.90$; $C_i^{\mathrm{cor,M}}$: $F_{2,75} = 11.1$, $P = 6.2 \times 10^{-5}$, $\eta^2 = 0.92$). However, as suggested in Fig. 4, the brain system that showed the most positive correlation with the age was the auditory network ($C_i^{\mathrm{wei,A}}$, Auditory − SMN: $t_{45} = 3.82$, $P = 0.00041$, $d = 1.14$; $C_i^{\mathrm{wei,A}}$, Auditory − Visual: $t_{42} = 3.34$, $P = 0.0018$, $d = 1.03$; $C_i^{\mathrm{wei,M}}$, Auditory − SMN: $t_{45} = 4.20$, $P = 0.00013$, $d = 1.25$; $C_i^{\mathrm{wei,M}}$, Auditory − Visual: $t_{42} = 4.17$, $P = 0.00015$, $d = 1.29$). These results are consistent with those shown in section 3.6. In other words, the assoiation between the clustering coefficients for weighted networks and the age is confounded by that between the node strength and the age. In contrast, the proposed clustering coefficients measure the effect of local clustering on the age without being confounded by the node strength.

References

[1] F. Heider, Attitudes and cognitive organization, J. Psychol 21 (1946) 107–112.

[2] S. Wasserman, K. Faust, Social Network Analysis, Cambridge University Press, New York, NY, 1994.

[3] M. Szell, R. Lambiotte, S. Thurner, Multirelational organization of large-scale social networks in an online world, Proc. Natl. Acad. Sci. USA 107 (2010) 13636–13641.

[4] G. Facchetti, G. Iacono, C. Altafini, Computing global structural balance in large-scale signed social networks, Proc. Natl. Acad. Sci. USA 108 (2011) 20953–20958.

[5] G. Costantini, M. Perugini, Generalization of clustering coefficients to signed correlation networks, PLOS ONE 9 (2014) e88669.

[6] B. Weaver, K. L. Wuensch, SPSS and SAS programs for comparing Pearson correlations and OLS regression coefficients, Behav. Res. Meth. 45 (2013) 880–895.

[7] D. A. Fair, A. L. Cohen, J. D. Power, N. U. F. Dosenbach, J. A. Church, F. M. Miezin, B. L. Schlaggar, S. E. Petersen, Functional brain networks develop from a "local to distributed" organization, PLOS Comput. Biol. 5 (2009) e1000381.

[8] J. D. Power, A. L. Cohen, S. M. Nelson, G. S. Wig, K. A. Barnes, J. A. Church, A. C. Vogel, T. O. Laumann, F. M. Miezin, B. L. Schlaggar, S. E. Petersen, Functional network organization of the human brain, Neuron 72 (2011) 665–678.

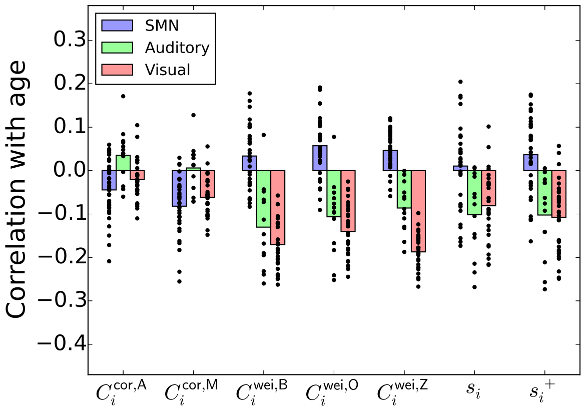

Figure C.1: Pearson correlation coefficient between a nodal index and the age, averaged over the ROIs in the SMN, auditory network or visual network. The circle represents the correlation coefficient value for a single node.
